# Comparative pharmacokinetics of maxacalcitol in healthy Taiwanese and Japanese subjects

**DOI:** 10.1016/j.heliyon.2020.e03538

**Published:** 2020-03-10

**Authors:** Ming-Che Liu, Feng-Yi Chou, Yi-An Chien, Yen-Ju Chen, Masaichi Abe, Koki Furusho, Shunji Matsuki

**Affiliations:** a Clinical Research Center, Taipei Medical University Hospital, Taiwan; b Chugai Pharma Taiwan Ltd., Taiwan; c Chugai Pharmaceutical Co. Ltd., Tokyo, Japan; d SOUSEIKAI Sugioka Memorial Hospital, Clinical Research Center (current SOUSEIKAI Fukuoka Mirai Hospital Clinical Research Center), Japan

**Keywords:** Maxacalcitol, Pharamcokinetics, Phase I study, Oxarol, Direct comparison study, Physical chemistry, Pharmaceutical science, Musculoskeletal system, Renal system, Pharmacology

## Abstract

Pharmacokinetic studies of maxacalcitol in healthy Taiwanese subjects have been conducted. This study to compare the pharmacokinetic properties of maxacalcitol in healthy Taiwanese and Japanese subjects. Healthy male Taiwanese subjects (n = 24) and healthy male Japanese subjects (n = 24) were enrolled in separate single-center and received a single intravenous dose of 1.25, 2.5 and 5 μg maxacalcitol. Male subjects were exclusively employed in the study due to the first administration of maxacalcitol to Taiwanese.

Serum samples were collected for up to 72 h for pharmacokinetic analysis, and safety was assessed. Exposures to maxacalcitol as mean C_5_ and AUC_inf_ appeared to increase with increase of doses in Taiwanese subjects (C_5_: 74.0, 159, and 321 pg/mL; AUC_inf_: 473, 763, and 1460 h･pg/mL) and Japanese subjects (C_5_: 92.9, 174, and 346 pg/mL; AUC_inf_: 312, 588, and 1040 h･pg/mL). After single bolus IV administration, linearity in maxacalcitol exposure was shown over the dose range of 1.25–5 μg in both Taiwanese and Japanese male healthy subjects. C_5_ of maxacalcitol was slightly lower (85%) in Taiwanese compared with that in Japanese and AUC_inf_ of maxacalcitol in Taiwanese subjects was contrarily 15.0 (41.6%) higher than that in Japanese subjects, resulted in not much difference in pharmacokinetics of maxacalcitol between Taiwanese and Japanese. Moreover, maxacalcitol was well tolerated in both healthy Taiwanese and Japanese subjects.

## 1 Introduction

**Figure 1:**
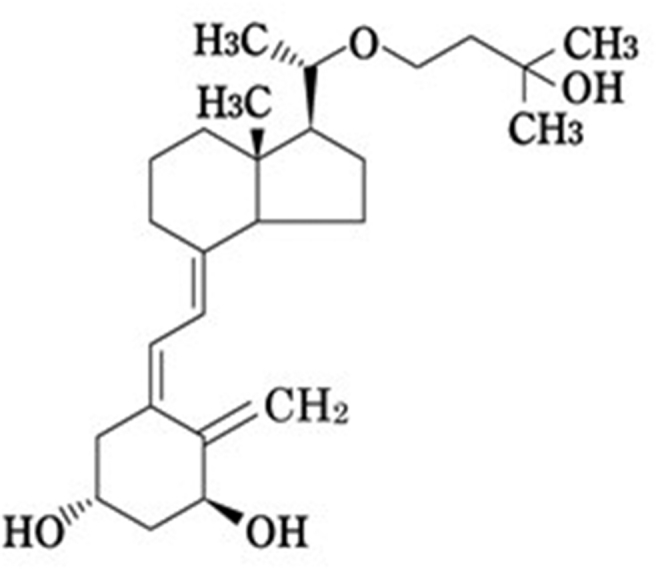
Chemical structure of maxacalcitol.

Maxacalcitol (OXAROL®) is chemically designed as (+)-(5Z, 7E)-(1S, 3R, 20S)-20-(3-Hydroxy-3-methylbutyloxy)-9, 10-secopregna-5, 7, 10(19)-triene-1, 3-diol. The empirical formula is C_26_H_42_O_4_ and its molecular weight is 418.62 [1]. The chemical structure is represented in Figure 1 [2].

Maxacalcitol is an active Vitamin D_3_ derivative which is indicated for secondary hyperparathyroidism (SHPT) in patients receiving maintenance dialysis. The recommended adult dose of maxacalcitol is 2.5 μg–10 μg as administered by intravenous (IV) infusion three times per week immediately before completion of dialysis. If there is no improvement in serum parathyroid hormone (PTH) level, the dose could be increased up to a maximum of 20 μg, with monitoring for the development of hypercalcemia [3].

Pharmacokinetic studies of maxacalcitol in healthy Taiwanese subjects have not been conducted, before introducing it to the Taiwan market. The objective of this study was to compare the pharmacokinetics and safety of maxacalcitol when administered as a single dose of 1.25–5 μg to healthy Taiwanese and Japanese subjects.

## 2 Materials and methods

### 2.1 Study design

The health authority in Taiwan (Taiwan Food and Drug Administration, TFDA) required at least 10 subjects in the PK study according to Regulations for Registration of Medicinal Products [4]. Twenty-four (24) Taiwanese and 24 Japanese were planned to be enrolled to evaluate the PK of maxacalcitol. The sample size was not statistically-calculated, but was based on the feasibility of the study.

This study was an open-label, two-center study to evaluate PK of maxacalcitol in Taiwanese and Japanese male healthy volunteers. There were three dose groups (Treatment A, B and C) in this study, with eight Taiwanese and eight Japanese subjects in each group. The study was conducted in Taiwan and Japan, in order to enroll Taiwanese and Japanese male healthy volunteers, respectively. Treatment A (a single dose of maxacalcitol 1.25 μg), Treatment B (a single dose of maxacalcitol 2.5 μg) or Treatment C (a single dose of maxacalcitol 5 μg) was administered intravenously as a bolus injection to subjects in groups A, B or C, respectively, followed by one-week observation. The last observation was performed seven days (±one day) after the day of dosing.

For healthy subject selection, the following inclusion criteria had to be met by all subjects to be eligible for the study:

1. Male subject whose age is between 20 to 45 years with healthy condition at the time of consent acquisition.
2. Subject whose body mass index (BMI) is between 18.5 kg/m^2^ to 25.0 kg/m^2^.

The study was conducted in compliance with the Declaration of Helsinki and the Guidelines for Good Clinical Practice. The study was also ethically reviewed and then approved before the initiation of the study by following institutional review committees at each site.

- TMU-Joint Institutional Review Board for Taipei Medical University Hospital (ethical number, N201510041)
- Hakata Clinic Institutional Review Committee for SOUSEIKAI Sugioka Memorial Hospital (ethical number, 1548CP)

Written informed consent was obtained from all subjects prior to participation in the study.

### 2.2 Drug administration

Eligible subjects were hospitalized for 3 nights, starting from the day before administration of study drug. On the day of drug administration, subjects received a single IV dose of maxacalcitol (1.25–5 μg).

#### 2.2.1 Treatment A

Maxacalcitol 2.5 μg (maxacalcitol 2.5 μg/ampule, 1 mL), a single bolus IV administration of maxacalcitol 1.25 μg (0.5 mL).

#### 2.2.2 Treatment B

Maxacalcitol 2.5 μg (maxacalcitol 2.5 μg/ampule, 1 mL), a single bolus IV administration of maxacalcitol 2.5 μg (1 mL).

#### 2.2.3 Treatment C

Maxacalcitol 5 μg (maxacalcitol 5 μg/ampule, 1 mL), a single bolus IV administration of maxacalcitol 5 μg (1 mL).

### 2.3 Blood sampling

Serum samples were collected at designated times for pharmacokinetic analysis. For the subjects, blood samples were collected before drug administration (0 h) and at 5, 10, 20, 30, 45 min, 1, 1.25, 1.5, 2, 2.5, 4, 8, 12, 24 and 48 h after dosing. 7 mL of blood was collected into plain vacutainers®. Following centrifugation, the serum supernatants were stored below - 20 °C until assay.

### 2.4 Sample analysis

Serum concentrations of maxacalcitol were determined by a sensitive and specific LC-MS/MS method. The samples were analyzed using a 500-μL aliquot and solid phase extraction. Extracts were injected into an LC system, and then detection was performed using a triple quadrupole mass spectrometer operated in the positive mode. Data were collected using selected ion monitoring. The lower limits of quantitation (LLOQ) for maxacalcitol was 10.0 pg/mL in serum. The method was validated in accordance with the recent regulatory guidelines. All samples were analyzed by Bozo Research Center, Tsukuba Research Institute, 8 Okubo, Tsukuba-shi, Ibaraki, 300-2611, Japan.

### 2.5 Pharmacokinetic analysis

Calculation of pharmacokinetic parameters was done by a non-compartmental method using SAS® (version 9.2, SAS Institute., NC, USA). The estimated PK parameters of maxacalcitol were back-extrapolated initial serum concentration (C_0_), drug concentration at 5 min (C_5_), maximum serum drug concentration (C_max_), time to maximum serum drug concentration (T_max_), area under the serum concentration-time curve from time zero to time of the last measurable concentration (AUC_last_), area under the serum concentration-time curve from time zero to infinity (AUC_inf_), percentage AUC extrapolated (AUC_extrap%_), elimination rate constant (k_el_), elimination half-life (t_1/2_), mean residence time (MRT), total body clearance (CL), volume of distribution (V_d_), and volume of distribution at steady state (V_d,ss_).

All pharmacokinetic parameters were summarized descriptively using mean, standard deviation values.

The linearity of maxacalcitol exposure (C_5_, AUC_last_ and AUC_inf_) was evaluated by power model analysis in which log(y) = α + β•log (dose), where β was the slope, α was an intercept, and y represented the PK parameter. Dose proportionality was concluded when the 90% confidence interval (CI) constructed on the estimate of slope parameter β included 1. Ethnic difference of PK parameters (C_5_, AUC_last_ and AUC_inf_) between Taiwanese and Japanese was evaluated by the point estimate and two-sided 90% CI of the geometric mean ratio (Taiwanese/Japanese) with combined group data.

### 2.6 Safety assessments

AEs were summarized by Medical Dictionary for Regulatory Activities (MedDRA), and the number of the events was categorized by System Organ Class (SOC) and the Preferred Term (PT). For events that the causality could not be excluded, they were separately listed as adverse drug reactions (ADRs). Laboratory data, marked abnormalities and clinical relevant change from baseline were summarized and evaluated. Physical examination, vital signs, and their changed from baseline were summarized by ethnicity and treatment. QTcF of standard 12-lead ECG were summarized based on criterion of ICH E14.

## 3 Results

### 3.1 Pharmacokinetics

**Figure 2:**
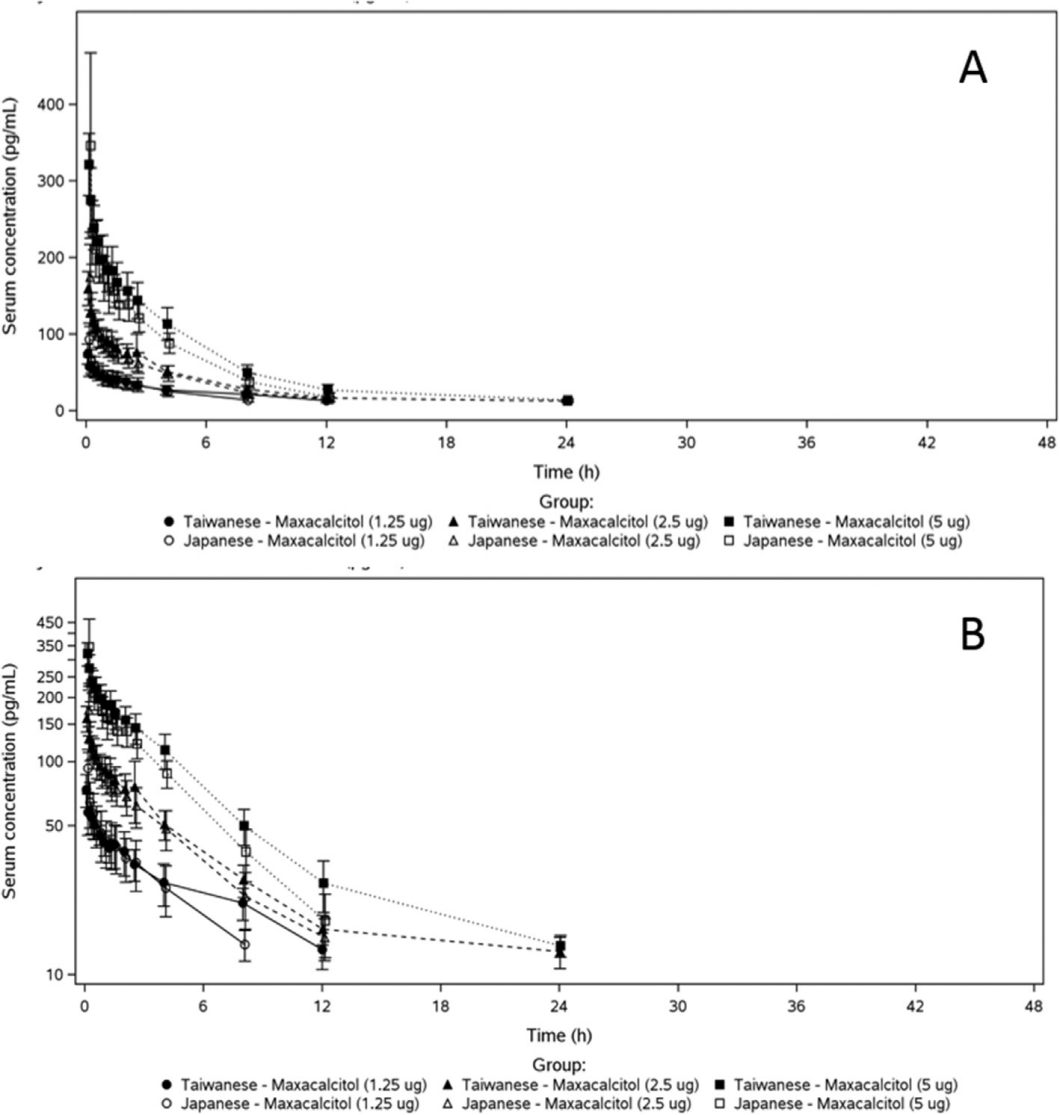
Mean serum concentration-time profiles of maxacalcitol (A) linear scale and (B) semi-log scale following single IV dose administration (1.25, 2.5 and 5 μg) in healthy Taiwanese and Japanese subjects.

A total of 48 healthy male subjects (Taiwanese, n = 24; Japanese, n = 24) completed the study, and were included in this analysis. The summary of demographic characteristics of all subjects are summarized in Table 1. Mean serum concentration-time profiles of maxacalcitol following single IV dose administration (1.25, 2.5 and 5 μg) in healthy Taiwanese and Japanese subjects were shown in Figure 2.

**Table 1:** Summary demographics of study populations.

Ethnicity	Taiwanese			Japanese		
Group	A (1.25 μg)	B (2.5 μg)	C (5 μg)	A (1.25 μg)	B (2.5 μg)	C (5 μg)
Variable^a^	N = 8	N = 8	N = 8	N = 8	N = 8	N = 8
Age (yr)	26.4 (6.2)	29.8 (7.2)	27.1 (5.4)	23.9 (3.0)	24.3 (4.3)	26.3 (4.7)
Weight (kg)	67.34 (8.98)	68.83 (9.30)	68.61 (4.33)	67.08 (7.04)	67.85 (7.39)	64.51 (9.63)
Height (cm)	174.09 (6.59)	174.89 (6.09)	172.74 (3.79)	171.99 (6.96)	176.66 (5.03)	171.21 (4.10)
BMI (kg/m^2^)	22.15 (1.97)	22.43 (2.02)	23.01 (1.54)	22.66 (1.77)	21.74 (2.26)	21.96 (2.78)

a All values were mean (SD).

The mean C_5_ and AUC_inf_ of maxacalcitol appeared to increase with increasing dose in Taiwanese subjects (C_5_: 74.0, 159, and 321 pg/mL; AUC_inf_: 473, 763, and 1460 h･pg/mL) and Japanese subjects (C_5_: 92.9, 174, and 346 pg/mL; AUC_inf_: 312, 588, and 1040 h･pg/mL). Following IV administration of a single dose (1.25–5 μg) of maxacalcitol, the mean serum C_5_ concentrations of maxacalcitol were lower in the healthy Taiwanese subjects than that of Japanese subjects, contrary AUC_inf_ and AUC_last_ were higher in Taiwanese than Japanese. The mean t_1/2_ of maxacalcitol were longer in Taiwanese subjects than in Japanese subjects at doses of 1.25 μg (431 vs. 240 min), 2.5 μg (828 vs. 235 min), and 5 μg (316 vs. 205 min) (Table 2).

**Table 2:** Pharmacokinetic parameters of maxacalcitol in healthy Taiwanese and Japanese subjects.

Ethnicity	Taiwanese			Japanese		
Group	A (1.25 μg)	B (2.5 μg)	C (5 μg)	A (1.25 μg)	B (2.5 μg)	C (5 μg)
Parameter^a^	N = 8	N = 8^b^	N = 8	N = 8^c^	N = 8	N = 8
C_0_ (pg/mL)	96.7 (25.1)	201 (39.9)	378 (59.0)	160 (88.0)	248 (114)	570 (401)
C_5_ (pg/mL)	74.0 (13.1)	159 (22.1)	321 (40.5)	92.9 (21.2)	174 (42.5)	346 (121)
C_max_ (pg/mL)	74.7 (12.2)	159 (22.1)	321 (40.5)	110 (60.6)	174 (42.5)	349 (116)
T_max_ (h)	0.115 (0.0884)	0.0833 (0.00)	0.0833 (0.00)	0.0833 (0.0267)	0.0833 (0.00)	0.0938 (0.0295)
AUC_last_ (h･pg/mL)	333 (78.5)	703 (219)	1340 (250)	240 (78.1)	500 (127)	951 (142)
AUC_inf_ (h･pg/mL)	473 (98.0)	763 (178)	1460 (257)	312 (80.9)	588 (142)	1040 (167)
AUC_extrap%_ (%)	29.6 (8.48)	20.1 (14.1)	8.33 (1.81)	24.8 (9.04)	15.3 (2.86)	8.63 (3.33)
k_el_ (1/h)	0.102 (0.0251)	0.116 (0.0483)	0.138 (0.0352)	0.176 (0.0219)	0.180 (0.0260)	0.207 (0.0300)
t_1/2_ (min)	431 (116)	828 (1420)	316 (73.4)	240 (28.6)	235 (32.5)	205 (31.8)
MRT (h)	10.4 (2.95)	7.98 (1.86)	7.19 (1.48)	5.41 (0.659)	5.30 (0.782)	4.62 (0.812)
CL (mL/kg/h)	41.5 (10.6)	50.8 (18.1)	51.5 (9.10)	65.4 (24.2)	65.7 (13.3)	77.5 (15.7)
V_d_ (mL/kg)	408 (58.8)	380 (71.2)	383 (76.6)	371 (126)	363 (38.7)	373 (46.0)
V_d,ss_ (mL/kg)	408 (68.4)	381 (61.5)	365 (81.5)	348 (114)	341 (39.0)	349 (41.5)

a All values were mean (SD).

b N = 7 for AUCinf, MRT, CL, Vd, and Vd,ss.

c N = 6 for C5.

Results of power model analysis revealed that the 90% CIs of the slopes for AUC_last_ in Taiwanese subjects (90% CI: 0.86–1.16) and Japanese subjects (90% CI: 0.84–1.23) both interrupted 1.00 (Table 3), indicating linearity of maxacalcitol exposure was shown over the dose range of 1.25–5 μg in both Taiwanese and Japanese subjects.

**Table 3:** Estimation of linearity pharmacokinetics of maxacalcitol.

Estimate (90% CI) of Slope	Taiwanese	Japanese	All
Parameter	N = 24^a^	N = 24^b^	N = 48^c^
C_5_ (pg/mL)	1.06 (0.97–1.16)	0.92 (0.73–1.12)	1.00 (0.90–1.11)
AUC_last_ (h･pg/mL)	1.01 (0.86–1.16)	1.04 (0.84–1.23)	1.02 (0.88–1.16)
AUC_inf_ (h･pg/mL)	0.81 (0.69–0.94)	0.89 (0.74–1.05)	0.85 (0.73–0.98)

a N = 23 for AUC_inf_.

b N = 22 for C_5_.

c N = 46 for C_5_ and N = 47 for AUC_inf_.

Based on the estimated geometric mean ratios (Taiwanese/Japanese) by ANOVA analysis (Table 4), C_5_ of maxacalcitol decreased by 15.0% with 90% CI range 0.627–1.152, while AUC_last_ and AUC_inf_ of maxacalcitol increased by 41.4% and 41.6% with 90% CI ranges from 1.031 to 1.938 and 1.083 to 1.850, respectively, as compared Taiwanese subjects with Japanese subjects.

**Table 4:** Estimation of ethnic difference of maxacalcitol.

Parameter	N	Geometric Mean Ratio (Taiwanese/Japanese)	
		Estimate	90% CI
C_5_ (pg/mL)	46	0.850	0.627–1.152
AUC_last_ (h･pg/mL)	48	1.414	1.031–1.938
AUC_inf_ (h･pg/mL)	47	1.416	1.083–1.850

### 3.2 Safety and tolerability

No clinically significant abnormal Ca, P, intact-PTH, and 1,25(OH)_2_D_3_ values, vital signs, physical examination, and ECG results were observed in Taiwanese and Japanese subjects in this study. Following single IV administration of maxacalcitol 1.25 μg, blood potassium increased at approximately 24 h post dose and recovered next day without treatment required in 37.5% (3/8 subjects) Taiwanese subjects. There were no deaths, other SAEs, other significant AEs or withdrawals due to AEs during the study.

## 4 Discussion

Chugai Pharmaceutical Co., Ltd. created MAXACALCITOL in response to the discovery that intravenous active vitamin D_3_ reduces high levels of serum PTH by Slatopolsky et al in 1984 [5] and Tsukamoto et al in 1989 [6]. Maxacalcitol was created by replacing the 22-carbon of calcitriol [1α,25(OH)_2_D_3_]), the active form of vitamin D, with an oxygen atom. This resulted in a drug that has a short blood half-life due to its weak binding affinity for vitamin D-binding protein and that suppresses PTH with a weak calcemic effect *in vitro* and *in vivo* [7]. Thus, the PK profiles were investigated in a single dose manner for the sake of a short half-life in blood with a long dosing interval (three times per week) [3].

Maxacalcitol is metabolized by CYP24 and CYP3A4 [8] whose ethnic differences have not been reported. Vitamin D receptor as target binding receptor of maxacalcitol and other vitamin D analogues also have not been reported that it has ethnic differences in East Asian ethnic groups [9, 10, 11]. In addition, the operational technic and experiences of haemodialysis in Taiwan are the same with those in Japan. By taking these facts into consideration, maxacalcitol doesn't have significant sensitive factors on pharmacodynamics, pharmacokinetics and its clinical use.

During clinical development of maxacalcitol, it was shown that the drug appreciably suppresses PTH when used to treat SHPT in patients on maintenance haemodialysis and that this inhibitory effect can be maintained by long-term treatment [12]. The results also showed that maxacalcitol corrects abnormally increased bone turnover in bone disease associated with SHPT. The most common adverse drug reaction (ADR) was slightly increased serum calcium (mild hypercalcemia), but this was able to be avoided by measures such as adjusting the dose. The results therefore showed that as well as being a basic therapy for suppressing PTH secretion in patients with SHPT, maxacalcitol also corrects bone disease in these patients.

It was concluded that maxacalcitol is a very useful drug medically as it has great clinical significance in the conservative treatment of SHPT and can also be expected to bring about improvement in patients with severe SHPT for whom surgical parathyroidectomy has previously been the only treatment option. Against this background, marketing authorization was obtained for maxacalcitol in 2000 as the first injected active vitamin D_3_ formulation in Japan, for the indication of “secondary hyperparathyroidism on maintenance dialysis”. Maxacalcitol is now widely used in Japan to treat SHPT and has been prescribed to an estimated 288,000 patients during the regulatory-required re-examination period (over 16 years).

Application for re-examination for this indication was filed in 2006 based on the results of drug use surveillance and other postmarketing surveillance conducted during the re-examination period. Notification of the re-examination results was received in 2008 and safety and efficacy were confirmed.

In Taiwan, SHPT patients were treated according to the KDIGO guide line and the Taiwan Chronic Kidney Disease Clinical Guidelines [13]. Oral active Vitamin D_3_ is used for the SHPT treatment but hypercalcemia and drug compliance are concerns. It is expected that maxacalcitol will contribute to the SHPT treatment in Taiwan with lower safety risks and better compliance than oral administration.

Chugai conducted this study to clarify the ethnic differences between Taiwanese and Japanese. After single IV administration, linearity of maxacalcitol exposure was shown over the dose range of 1.25–5 μg in both Taiwanese and Japanese male healthy subjects. C_5_ of maxacalcitol was slightly lower in Taiwanese compared with in Japanese and AUC_inf_ of maxacalcitol in Taiwanese subjects was 41.6% higher than that in Japanese subjects, resulted in not much difference in pharmacokinetics of maxacalcitol between Taiwanese and Japanese (Table 4).

A t_1/2_ of maxacalcitol was longer in Taiwanese subjects compared to Japanese subjects although the subject background and heart rate as vital sign, which may affect to hepatic blood flow rate, was similar in both subject groups. The baseline 1, 25(OH)_2_VD_3_ values were actually higher in Japanese than Taiwanese subjects in this study [14]. A difference in expression levels of relevant metabolic enzyme CYP24 or CYP3A4 at baseline or observation period of the study might have had impacts on pharmacokinetic property of maxacalcitol in subjects, however, these assessments were not done in the study.

Serum potassium values in three Taiwanese subjects were increased on Day 2 at approximately 24-hour post-dose and then returned to a normal range on Day 3 which was approximately 48-hour post-dose. Extent of changes in serum potassium level were extraordinary (6 or more mmol/L) as physiological electrolyte status, while the other laboratory test values were stable on those measurement points. There was also no change in ECG observation. Considering there was a little bit of increase in serum LDH, WBC or platelets level in association with serum potassium increase, there might have been “pseudohyperkalemia” caused by the processes of serum laboratory test sample preparation [15].

## 5 Conclusion

Judging by these pharmacokinetic and safety results from Taiwanese and Japanese subjects, although no new safety findings were observed in Taiwanese subjects, the exposures to maxacalcitol in Taiwanese were higher (41.4% for AUC_inf_ and 15.0% for C_max_) than those in Japanese.

## Declarations

### Author contribution statement

M. Abe and S. Matsuki: Conceived and designed the experiments; Analyzed and interpreted the data; Wrote the paper.

M. Liu: Conceived and designed the experiments; Performed the experiments; Contributed reagents, materials, analysis tools or data; Wrote the paper.

F. Chou: Performed the experiments; Contributed reagents, materials, analysis tools or data.

Y. Chien, Y. Chen and K. Furusho: Performed the experiments; Contributed reagents, materials, analysis tools or data.

K. Furusho: Conceived and designed the experiments; Performed the experiments; Analyzed and interpreted the data; Contributed reagents, materials, analysis tools or data; Wrote the paper.

### Funding statement

The study was sponsored by Chugai Pharmaceutical Co. Ltd in Japan.

### Competing interest statement

Ming-Che Liu MD, Feng-Yi,Chou, Shunji Matsuki MD and Koki Furusho have no conflict of interest to disclose. Yi-An Chien and Yen-Ju Chen are employees of Chugai Pharma Taiwan Ltd., and Masaichi Abe is an employee of Chugai Pharmaceutical Co. Ltd.

### Additional information

No additional information is available for this paper.
